# Editorial: Women in infectious diseases epidemiology

**DOI:** 10.3389/fepid.2023.1351528

**Published:** 2024-01-10

**Authors:** Jaishree Raman

**Affiliations:** ^1^ Centre for Emerging Zoonotic and Parasitic Diseases, National Institute of Communicable Diseases (NICD), Johannesburg, South Africa; ^2^ Wits Research Institute for Malaria, Faculty of Health Sciences, University of Witwatersrand, Johannesburg, South Africa; ^3^ UP Institute for Sustainable Malaria Control, Faculty of Health Sciences, University of Pretoria, Pretoria, South Africa

**Keywords:** epidemiology, infectious diseases, women, COVID-19, congenital syphilis, fungemia, malaria

**Editorial on the Research Topic** Women in infectious diseases epidemiology

Despite concerted efforts to increase the representation of women and young girls activity engaged in science, technology, engineering, and mathematics (STEM), they remained significantly underrepresented on the global stage. Currently, less than 30% of STEM researchers worldwide are women. To showcase women from across the globe leading research on the epidemiology of infectious diseases, Frontiers in Epidemiology, launched this inaugural Research Topic “Women in Infectious Disease Epidemiology”. Women researchers included in this six-article Research Topic are based in Africa, Europe, North America, the Middle East, and South America and work on a variety of infectious diseases ranging from the novel coronavirus, SAR-CoV-2, to congenital syphilis.

The first article in this Research Topic by Chaipitakporn et al., investigated the impact of four air pollutants and certain sociodemographic factors (county population density, age, poverty, and education levels, percentage of Hispanic and African Americans) on COVID-19-related morbidity and mortality in American counties with population densities of ≥100,000 during the first pandemic wave and subsequent surge. Only, being over 65 was associated with infections and fatality in both waves. Population density and education level were only associated with infection rate during the first wave. These two factors together with poverty and nitrogen oxide levels were associated with fatalities only in the first wave. This study demonstrated that risk factors may change over the course of a pandemic, calling for routine assessment of risk factors to inform prevention and mitigation strategies as pandemics progress.

The second COVID-19-related article in this Research Topic by Vremera et al., reports on the immune status of vaccinated individuals, 65 years or older, living in Romanian residential care homes. Serum collected from 635 participants, two to 12 weeks after vaccination was analysed using a chemiluminescent immunoassay to determine anti-spike antibody levels. Protective levels of the anti-spike antibodies were found in 99% of the participants, despite 55% of the participants reporting at least one comorbidity. Individuals with a history of COVID-19 infection had higher levels of protective antibodies. The data from this study strongly supports the vaccination of the elderly against COVID-19, irrespective of the presence of commodities, disability, or history of COVID-19 infection.

The third and final article on COVID-19 in this Research Topic is by Nolna et al., who studied the association between HIV status and susceptibility to SARS-CoV-2 infection in Cameroon. Patients visiting HIV units of two healthcare facilities in Douala, Cameroon, were enrolled and assessed for the presence of anti-SARS-CoV-2 antibodies using an immunoassay. Information on viral load, antiretroviral treatment, and duration of infection was extracted from the hospital records for the participants living with HIV. No association between HIV status, viral load, antiretroviral treatment, or duration of infection was found. Data from this study suggest that people living with HIV, where the disease is controlled, are at no greater risk of SARS-CoV-2 infection compared to the general public.

Khateb et al., conducted a retrospective trend analysis of fungal infection among inpatients at the King Fahad Hospital in Medina, Saudi Arabia from 2013 to 2019. Information on causative agents, treatment prescribed, and patient demographics was obtained for 331 fungemia episodes from the hospital records and analyzed. Over the study period, fungemia episodes and the use of antifungal treatments increased significantly. Infections were more prevalent in women (62%), central blood samples incubated aerobically (55%), and individuals with renal disease (24%). While *Candida parasilosis* was the most prevalent yeast species, increasing levels of antimicrobial resistance were detected in *Candida albican* and *Candida glabrata*. This study highlights the need for the development and implementation of policies to effectively manage fungal infections and prevent resistance from spreading in Saudi Arabia.

Sustained cross-border malaria transmission poses a challenge to countries in southern Africa trying to eliminate malaria. Gwarinda et al., assessed malaria parasite population structure in four southern African countries; Eswatini, Mozambique, Namibia, and South Africa using microsatellite markers to identify factors that could be exploited to advance elimination efforts. Parasites from Namibia contained more unique microsatellite alleles compared to parasites from South Africa and Eswatini. In addition, polyclonal infections were most frequently detected in malaria infections from Namibia. Gene flow patterns suggested that most of the parasites circulating in the four study countries originated from Mozambique. This information can be used to guide the development of interventions to reduce the movement of parasites from Mozambique and advance the elimination efforts in the region.

The final paper in this Research Topic by Luz Vital et al., looks at the spatial distribution of congenital symphilis (CS) in Bahia, Brazil from 2009 until 2018. Prevalence data, obtained from the Notifiable Diseases Information and Live Birth Information Systems, were analyzed using the Global Moran Index 1 and Local Spatial Association Indicator to determine the spatial distribution of CS cases. There was a significant increase in CS cases over the study period, with four high-incidence municipalities identified, Alcobaca, Caraveles, Jandaira, and Teixweria de Freitas. Maternal factors associated with CS included being 20–29 years of age, not having completed primary school, and being black or multiracial. Following diagnosis, 69% of the mother and 81% of the partners, were not adequately treated. Data from the study confirms that CS is a major problem in Bahia, Brazil, and that urgent steps need to be taken to improve CS health awareness among young women of childbearing age and the delivery of CS treatment.

This Research Topic highlights the impressive and diverse range of research led by women researchers from across the globe who work in the epidemiology of infectious diseases. The work presented has the potential to improve the health and well-being of the affected communities and serve as a positive reminder that women can succeed and make a difference in infectious disease epidemiology.

